# Zeta Potential of Beta Zeolites: Influence of Structure, Acidity, pH, Temperature and Concentration

**DOI:** 10.3390/molecules23040946

**Published:** 2018-04-18

**Authors:** Xuan Liu, Päivi Mäki-Arvela, Atte Aho, Zuzana Vajglova, Vladimir M. Gun’ko, Ivo Heinmaa, Narendra Kumar, Kari Eränen, Tapio Salmi, Dmitry Yu. Murzin

**Affiliations:** 1 Laboratory of Industrial Chemistry and Reaction Engineering, Faculty of Science and Engineering, Johan Gadolin Process Chemistry Centre, Åbo Akademi University, FI-20500 Turku/Åbo, Finland; xuan.liu@abo.fi (X.L.); pmakiarv@abo.fi (P.M.-A.); atte.aho@abo.fi (A.A.); zuzana.vajglova@abo.fi (Z.V.); nkumar@abo.fi (N.K.); keranen@abo.fi (K.E.); tsalmi@abo.fi (T.S.); 2 Chuiko Institute of Surface Chemistry, National Academy of Sciences of Ukraine, 03164 Kiev, Ukraine; vlad_gunko@ukr.net; 3 National Institute of Chemical Physics and Biophysics, Akadeemia tee 23, 12618 Tallinn, Estonia; ivo.heinmaa@kbfi.ee

**Keywords:** zeolites, beta, zeta potential

## Abstract

Measurements of the zeta potential of solid heterogeneous supports are important for preparation of metal supported catalysts and for shaping zeolites into extrudates. In the current work, different types of heterogeneous support materials such as SiO_2_, Al_2_O_3_, and a range of beta zeolites of different silica- to-alumina ratio were analysed. It was observed that parameters such as temperature, pH and acidity significantly affect the zeta potential. In several instances, depending on the materials’ acidity and microstructure, maxima in zeta potential were observed. The solid materials were thoroughly characterized using XRD, SEM, EDX, TEM, nitrogen physisorption, Al-NMR and FTIR with pyridine before zeta potential measurements.

## 1. Introduction

The immense importance of catalysis in the chemical industry is manifested by the fact that roughly 85–90% of all chemical products have seen a (heterogeneous) catalyst during the course of their production. An important class of industrially applied heterogeneous catalysts includes various supported metal and metal or metalloid oxides, which activity and selectivity strongly depend on several factors, such as particle morphology and structure as well as support properties [1]. There are several methods to deposit an active phase on a support, including impregnation, deposition precipitation, strong electrostatic adsorption or colloidal stabilization of metal nanoparticles. In those methods, the surface properties are of importance to obtain the metal particles with appropriate cluster sizes. In fact, the metal particle size and its distribution are one of the most crucial properties of the catalysts. A relationship between the metal cluster size and relative difference between the points of zero charge (pzc, pH_pzc_) or isoelectric point (pH_IEP_) and pH of deposition was established [2].

As well-known ζ-potential is among the most important characteristics of colloidal systems being defined as the electric potential at the slipping plane which separates a mobile fluid from the fluid that remains attached to the surface [3,4,5]. Subsequently rheological behavior of molding masses influences the textural properties of extrudates, as mentioned by Foundas et al. [5]. Zeta potential gives information about the surface charge (including an immobile part of electric double layer, EDL) of the material being important not only for such applications as preparation of metal supported catalysts [1], but also for catalyst shaping [6,7]. For example, a correlation has been found between the difference in solution pH, zeolite pH_IEP_ and the relative particle attrition index measured in the Davison jet cap for FCC (fluid catalytic cracking) catalyst [8].

The influence of a binder on catalyst shaping and zeta potential was also considered in [7] linking zeta potential to the rheology of catalyst shaping [3,4,5]. The yield stress depends on the zeta potential through a complex relationship. An example of the influence of zeta potential on catalyst shaping was provided by Devyatkov et al. [7]. Shaping of zirconia together with the alumina binder was done at several values of pH. It was found that a decrease in pH favors a smoother flow and gives defect-free catalyst extrudates. At the same time the decrease in pH elevated the zeta potential negatively influencing the mechanical stability of extrudates. When zeta potential is far from zero the shear stress is lower making the shaping mass behavior more like a Newtonian-type. In some extreme cases this can result in so-called green bodies not capable of retaining their shapes. The pore structure of the formed zirconia-alumina extrudates [7] was unaffected by rheological parameters when the zeta potential is close to zero, while significant deviations from zero afforded more uniform pore size distributions. All these data show that the influence of surface properties and rheology should be taken into account while selecting the optimum zeta potential values for catalyst shaping.

The present paper deals with zeta potential measurements of catalyst supports such as silica and alumina. Additionally, a substantial effort was put on understanding zeta potential behavior of zeolites of different morphology, structure and acidity. Zeolites and metal-modified zeolites are widely used in industry, since they exhibit shape selectivity, their acidity can be tuned without changing the morphology and furthermore they can be easily regenerated and reused. Although, several publications can be found on zeta potential and surface charge measurements of aluminosilicates and zeolites [6,7,8,9,10,11,12,13,14,15,16,17,18,19,20,21,22,23,24,25,26,27,28,29,30,31], there is still a need for systematic information on the effect of support acidity [9] or temperature on the zeta potential.

Zeta potential measurements have been reported for several types of aluminosilicates, such as mica [10,11], muscovite [12], montmorillonite [13,14,15], zeolite Y [16], ZSM-5 [6], beta-zeolite [6,9], silicalite [9,17], bentonite [18], chlorite [19], kaolinite [13,20,21,22,23], kaolin [24], pyrophyllite, [25] and albite [26]. Additionally, electrophoretic surface charge was reported for attapulgite [27], electrophoretic mobility for montmorillonite [28], palygorskite [29] and bentonite [30], as well as surface charge for montmorillonite [31] and natural zeolite [32]. Typically, zeta potential decreases with increasing pH for such aluminosilicates as mica [10], chlorite [19], kaolinite [22], kaolin [24], pyrophyllite [25], albite [27], bentonite [18], and ZSM-5 [6]. Analogously, the electrophoretic mobility decreases with increasing pH for palygorskite [31]. There are also reports on the unusual behavior of zeta potential curves as a function of pH giving maxima [12,33] or irregular patterns [30].

A detailed description regarding determination of the zeta potential and particle sizes of pristine SiO_2_, Al_2_O_3_, and TiO_2_ has been provided in a review [34]. Modification of the pristine single oxide or mixed oxides by transition or noble metals can influence the zeta potential values [15]. The aim of the present research was to study the influence of pH on the zeta potential of the H form of H-beta zeolites with different SiO_2_/Al_2_O_3_ ratios. Conventional supports such as alumina and silica were used for reference purposes. Furthermore, the influence of temperature and concentration of tested catalysts in aqueous suspensions was also investigated. It should be noted that addition of any indifferent electrolyte (e.g., metal salts) strongly affects the electric double layer (EDL). Upon comparison of the electrophoretic behavior of different solids, a poorly controlled factor appears if the salinity of the suspension is not zero. In particular, an important factor which can influence in principle isoelectric point (IEP) is ionic strength, as IEP can change in the presence of such electrolytes as for example KOH [35]. This is important when the added electrolyte has a special affinity to the surface. Otherwise, the effect of the presence of electrolyte on IEP can be relatively minor, as evidenced for example for zeta potential of montmorillonite not changing the qualitative shape of the zeta potential curve as a function of pH [35]. To clarify this issue zeta potential measurements in this work were done predominantly in distilled water, while in some case the salinity of the suspensions was deviating from zero increasing the ionic strength of suspensions.

According to the best of our knowledge, there is only one publication on zeta potential studies for beta zeolites with varying SiO_2_/Al_2_O_3_ ratio [9]. It is also noteworthy to mention here that the research results reported are the first in-depth study of the influence of important parameters such as structure of support, pH, temperature, acidity and concentration on the values of zeta potential.

## 2. Results

### 2.1. XRD Results

**Figure 1:**
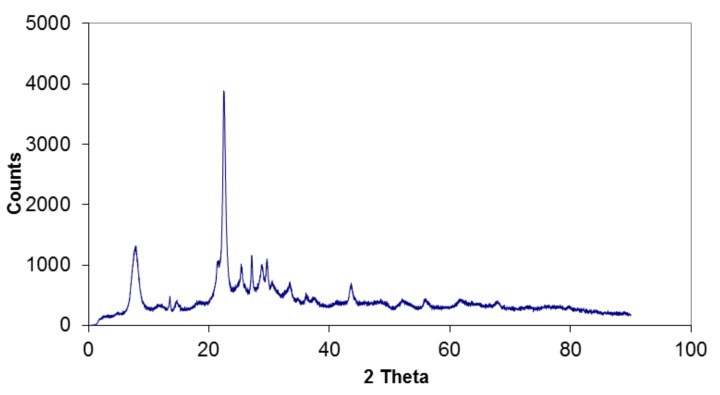
X-ray powder diffraction pattern of H-Beta-25 zeolite.

XRD measurements were performed to confirm the structures of zeolites used. The diffractogram of H-Beta shows clearly typical 2*θ* peaks of beta zeolite at 22.4° and 7.6° (Figure 1) [36] with a high degree of crystallinity.

### 2.2. Specific Surface Area and Morphology of Zeolites

Specific surface areas, *S*_BET_ (Table 1) of zeolites were determined from nitrogen adsorption isotherms. The values of *S*_BET_ of H-beta zeolites varied between 664 m^2^/g and 807 m^2^/g and appeared to not be correlated with the SiO_2_/Al_2_O_3_ ratio.

**Figure 2:**
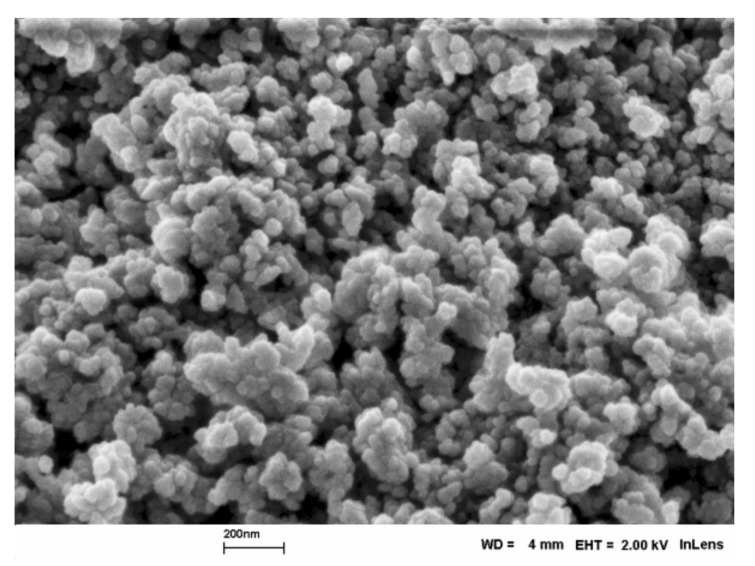
Scanning electron micrograph H-beta-25.

**Figure 3:**
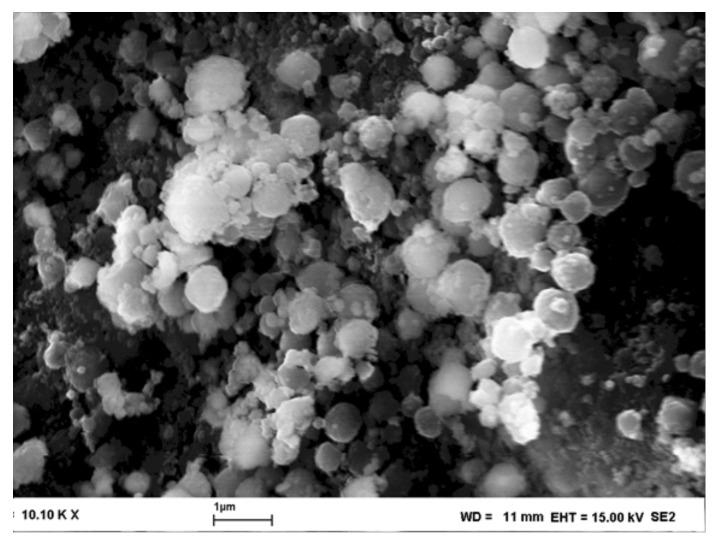
Scanning electron micrograph H-beta-300.

SEM image of H-beta zeolites are shown in Figure 2 and Figure 3. The morphology of H-beta-150 was similar to that seen in Figure 2.

The particles of H-beta zeolite are round shaped particles similar to the results reported in [38]. The size of H-beta-300 varied in the range of 0.4–1 μm, whereas the particles of H-beta-25 were much smaller, being in the range of 70–140 nm.

### 2.3. Acidity of Zeolites

The total amounts of Brønsted and Lewis acid sites in H-beta-300 are only 24% and 27% of those present in H-beta-25 (Table 2).

Acidity decreases as follows: H-beta-25 > H-beta-150 >> H-beta-300. According to ^27^Al MAS NMR spectra the total acidity of H-beta 25 and H-beta-150 are close to each other (Table 3).

**Figure 4:**
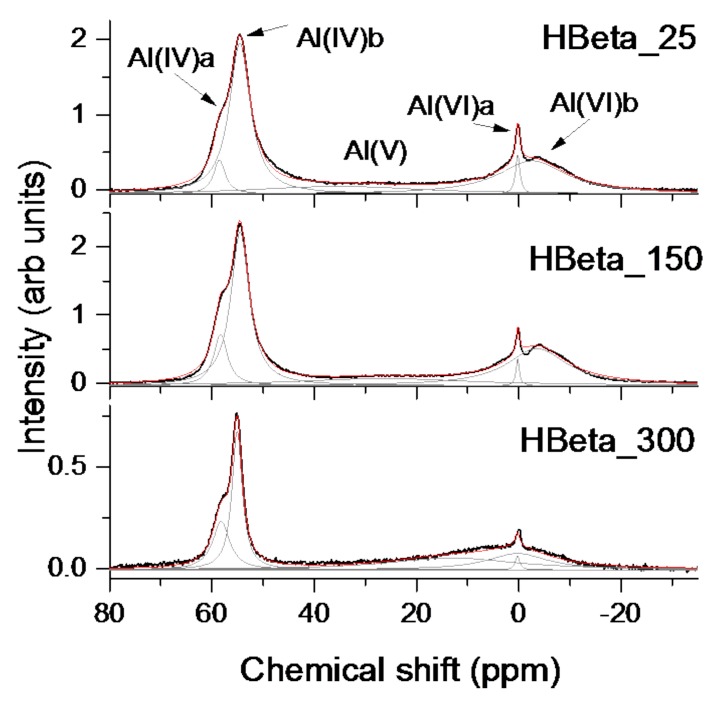
^27^Al-MAS NMR results from H-beta-25, H-beta-150, H-Beta-300 materials. Gray lines show the components of the spectra.

Extra framework Al species appear about 0 ppm and exhibit Lewis acidity, whereas framework Al species with Brønsted acidity display a signal at 60 ppm (Figure 4), which is linked to the O-coordination numbers of Al atoms [40]. Generally, the acidity of H-beta-25 and H-beta-150 is much higher than that measured for H-beta-300. This result follows from both pyridine desorption and analysis of ^27^Al-MAS NMR spectra.

### 2.4. Zeta Potential of Oxides and Zeolites

Zeta potential as a function of pH was determined for alumina, silica and H-beta. The main variables were sample concentration, SiO_2_/Al_2_O_3_ ratio and temperature.

#### 2.4.1. Zeta Potential for Individual Oxides

**Figure 5:**
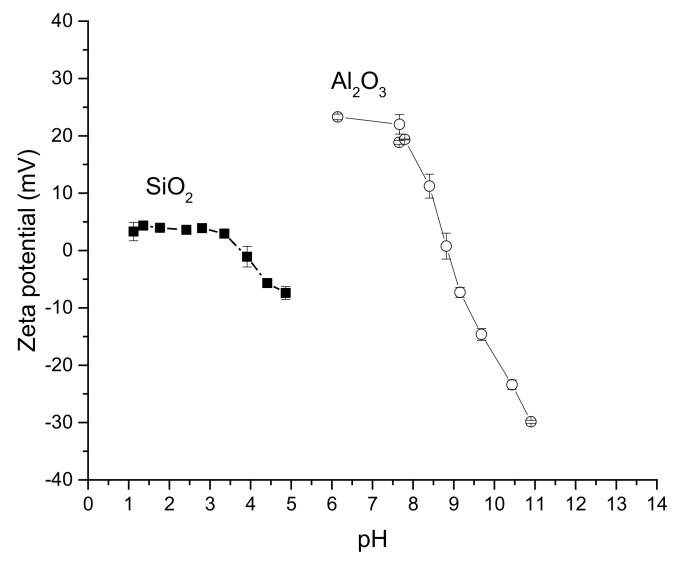
Zeta potential as a function of pH for Al_2_O_3_ (La Roche Versal alumina GL25) and for SiO_2_ (Merck silica gel Si-60). Conditions: 2 mg/mL, 25 °C.

Preliminary measurements of zeta potential were made for alumina and silica to check the data validity (Figure 5) [1]. Generally, under acidic pH the oxide surfaces are positively charged due to protonation of surface hydroxyl groups, whereas at high pH values deprotonation of terminal silanol and bridged oxygen for alumina occurs leading to negative surface charges. Zeta potential curves typically drop down with increasing pH (Figure 5). The IEP for silica and alumina were 3.9 and 8.8, respectively, being analogous to those reported in the literature [41]. Somewhat lower values for IEP (ca. 3) were also reported in the literature for silica [42].

**Figure 6:**
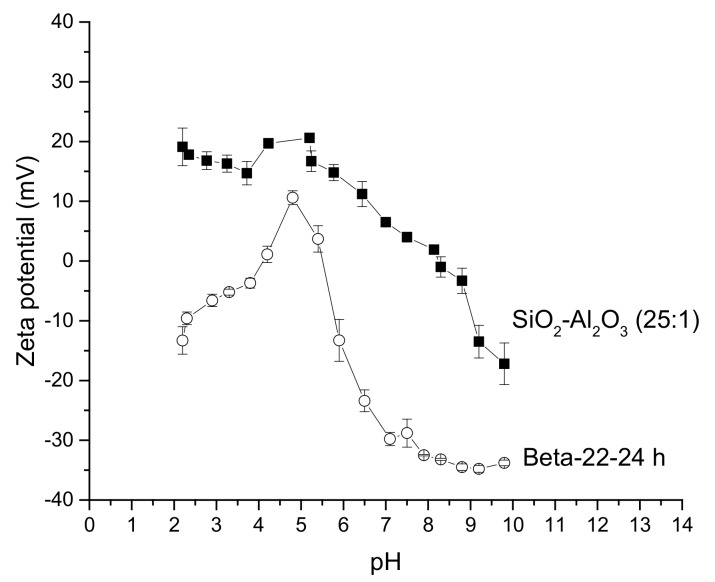
Zeta potential curves as a function of pH for a physical mixture SiO_2_-Al_2_O_3_ with a molar ratio of 25:1 and for an amorphous zeolite mixture with silica to alumina ratio of 22 aiming for Beta zeolite structure, synthesized for 24 h. Conditions: 2 mg/mL, 25 °C.

On the other hand, the zeta potential curve for a physical mixture of SiO_2_ (Merck, Darmstadt, Germany) and Al_2_O_3_ (UOP, Des Plaines, IL, USA) with the molar ratio of 25:1 resembled that of alumina, even if the alumina content in the mixture is rather low (Figure 6).

The value of IEP_c_ for this silica-alumina mixture was 8.1, whereas for pure SiO_2_ (Merck) and Al_2_O_3_ (UOP) it was respectively 3.8 and ca. 8.4. H-Beta-zeolite with silica to alumina ratio of 22 and synthesis time of only 24 h exhibited a maximum at pH 5 (Figure 6). The values pH_pzc1_ and pH_pzc2_ for this amorphous zeolite were 4.1 and 5.5, respectively. Figure 6 for illustration purposes contains error bars to indicate the measurement errors.

In general, it can be stated comparing the current zeta potential curves with those found in the literature for beta zeolite with different Si/Al ratios [9] that no maxima were found in [9] and the curves typically decrease with increasing pH.

#### 2.4.2. Effect of Temperature

**Figure 7:**
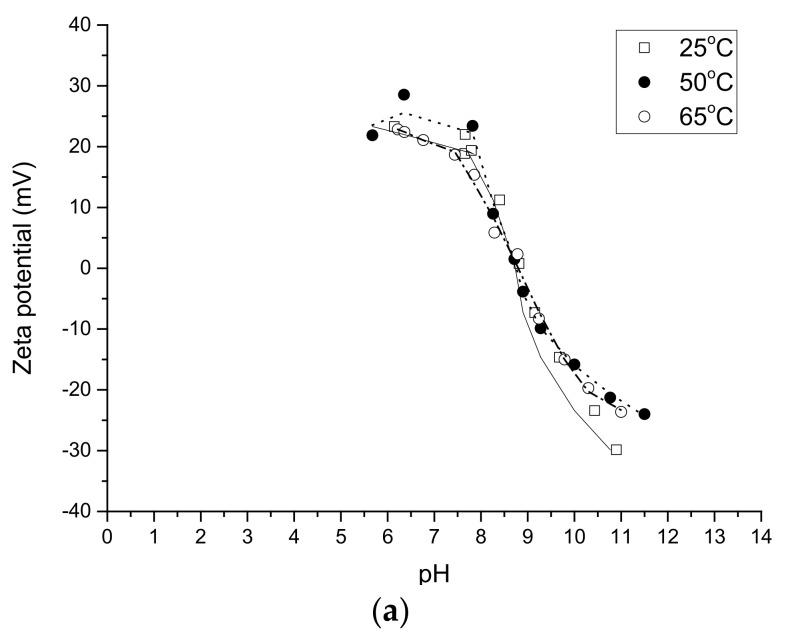

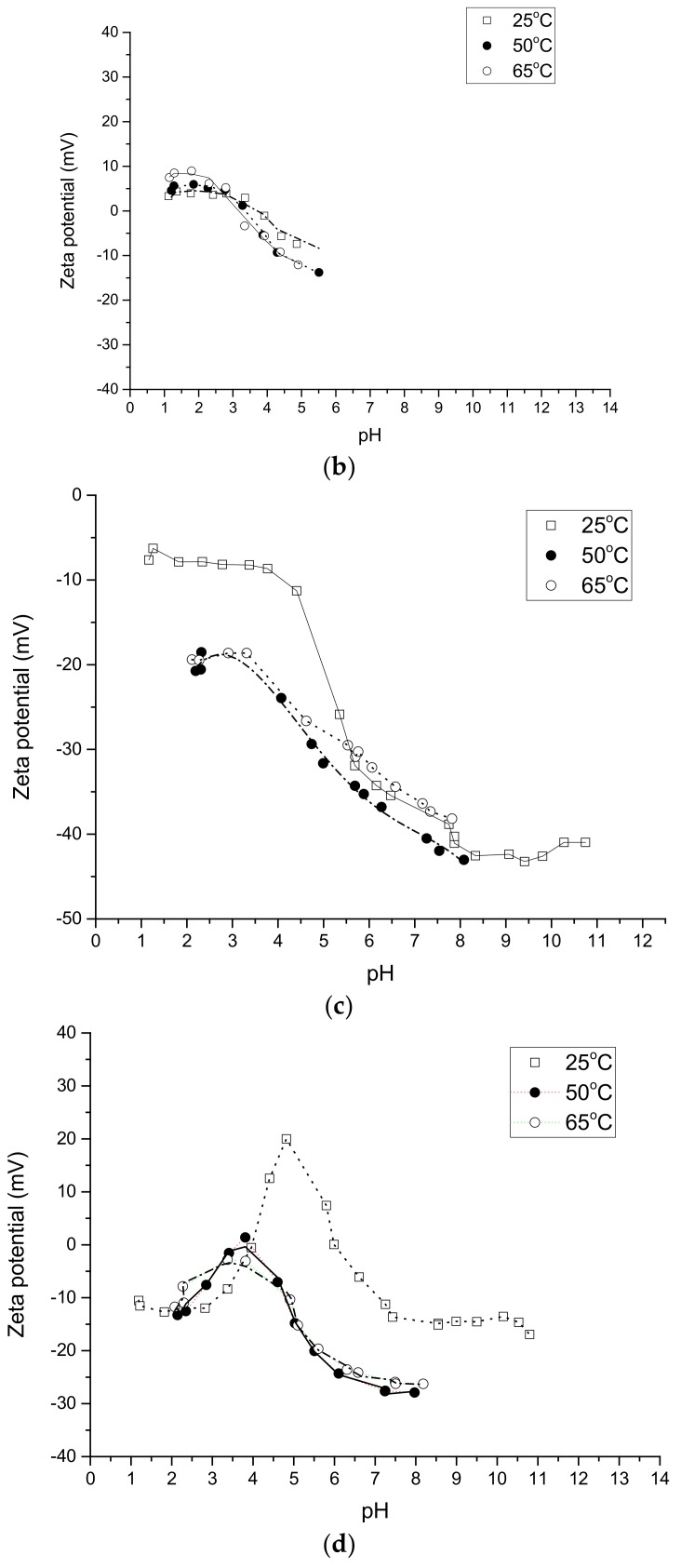
Effect of temperature on zeta potential for (**a**) alumina; (**b**) silica; (**c**) H-Beta-300; (**d**) H-Beta-25. Conditions: 2 mg/mL.

The effect of temperature on zeta potential for silica and alumina as well as for zeolites is shown in Figure 7. Zeta potential curves for γ-Al_2_O_3_ coincided and the point of zero charge for γ-Al_2_O_3_ has been constant (ca. 9.4) at all studied temperatures in the range 25–65 °C (Figure 7a). The zeta potential behavior vs. T can be a sign than protonation/deprotonation steps have negligible temperature dependence in the case of alumina (Figure 7a) and, to a certain extent, silica (Figure 7b). More precisely, the zeta potential values decreased for silica with increasing temperature (Figure 7b) from 3.8 (25 °C) to 3.1 (65 °C).

For zeolites the temperature effect was on the contrary very prominent (Figure 7c,d). Typically for H-beta-25 and H-beta-150 the maximum zeta potential values were the highest at 25 °C, whereas a larger decrease in the zeta potential values was observed at 50 °C and 65 °C. These two curves are located, however, relatively close to each other, as can be seen also from the maxima zeta potential values given for each H-beta zeolite (Table 4). Not surprisingly the zeta potential curves for H-beta-300 resembled those of silica. The highest initial zeta potential values for this zeolite type were achieved at 25 °C.

From the discussion above it can be hypothesized that the SiO_2_/Al_2_O_3_ ratio reflecting the amount of aluminum in the framework can be an important parameter in determining zeta-potential behavior. This ratio affects acidity of SiO_2_/Al_2_O_3_ which increases with decreasing alumina content. An increase of silica to alumina ratio is also accompanied by a stronger influence of the silica phase.

**Figure 8:**
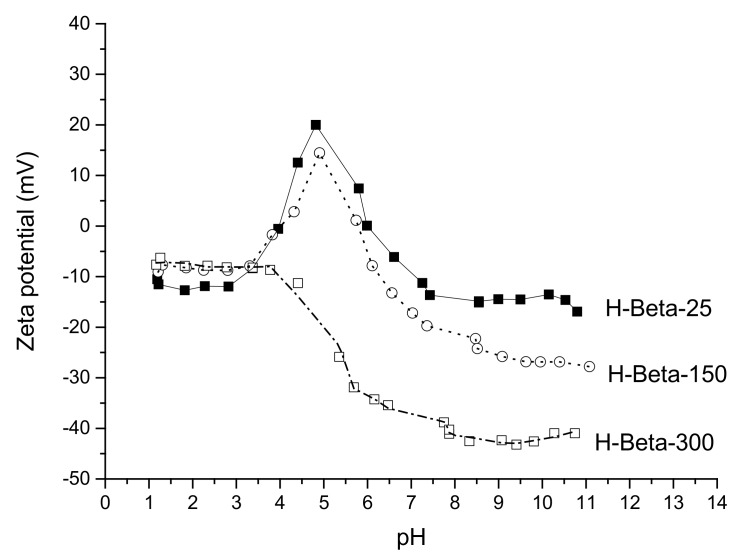
Zeta potential measurements of H-beta-25, H-beta-150 and H-beta-300 zeolite catalysts. Conditions: 2 mg/mL, 25 °C.

The effect of the SiO_2_/Al_2_O_3_ ratio on zeta potential was, therefore, tested for H-beta materials. The results show that for H-beta-25 and H-beta-150, pzc_1_ was at pH 4, whereas pzc_2_ for H-beta-150 and H-beta-25 was respectively at pH 5.7 and 6.0 (Figure 8). These two curves are relatively close to each other. It should be noted that almost the same values of isoelectric points (pzc_1_ and pzc_2_) were obtained for H-beta-25 at salinity of 0.01 or 0.05 KCl also with the maxima of zeta potential at pH 5. Contrary the zeta potential curve for H-beta-300 resembled a simpler behavior of silica without any maxima. For beta zeolites there are reports showing both the maximum [33] and smooth behavior [9] in the zeta potential dependence on pH.

It was observed that for H-beta-25, H-beta-150 and H-beta-300 zeolites, the zeta potential values were negative at 65 °C clearly highlighting the influence of temperature on zeta potential because the values obtained at 25 °C and 50 °C were positive.

H-Beta-25 zeolite catalyst exhibited the highest zeta potential at 25 °C. The influence of acidity (i.e., Brønsted and Lewis acid sites) on zeta potential was studied using H-beta-150 zeolite with a lower amount of acid sites than in H-beta-25. Furthermore, H-beta-150 at of 25 °C exhibited a lower zeta potential than H-beta-25, clearly indicating that the presence of Brønsted and Lewis acid sites, their number and strength influenced the values of zeta potential. It is noteworthy to point out here that, besides the amount of SiO_2_, Al_2_O_3_, SiO_2_/Al_2_O_3_, also the presence of Brønsted and Lewis acid sites can also influence the values of zeta potential and isoelectric point (Table 4). The negative values of the surface charge in a broad range of pH values for beta zeolite have been previously reported [33].

## 3. Discussion

It follows from the experimental data that the initial zeta potential value decreased with decreasing SiO_2_/Al_2_O_3_ ratio at low pH (Figure 8), while for beta-zeolites the initial values were rather similar. The maximum zeta potential value for beta zeolite was the highest with the lowest SiO_2_/Al_2_O_3_ ratio and decreasing with increasing SiO_2_/Al_2_O_3_ ratio. As a comparison with the literature, a maximum value for the zeta potential at a certain pH has been reported for kaolinite in the presence of metal salts, whereas a declining trend of zeta potential as a function of pH was achieved in pure water [21]. Furthermore, a maximum of the zeta potential was found for muscovite at pH 4–5 [12] similarly as in the current case for H-beta-25 and H-beta-150 at 25 °C. In the case of clay, the maximum in the zeta potential was higher, if the material had been aged at a low pH for a longer time. The complex behavior of zeta potential should be interpreted as done in [12] by taking into account partial dissolution of clay that occurred followed by adsorption of dissolved species at higher pH thus affecting the zeta potential values The structure of clays, however, differs from that of zeolites and thus it is difficult to relate their behavior in terms of the zeta potential curves.

For explanation of the complex behavior of zeta potential for zeolitic materials it should not be forgotten that the zeta potential and the values of surface charge represent some sort of lumped values and even for the case of the negative surface charge a negatively charged precursor can be deposited on the surface although in minor amounts [43]. Non-uniformity of surface sites in zeolites and thus presence of sites of different chemical nature and strength has been demonstrated by a range of techniques, including FTIR spectra of adsorbed pyridine [44], temperature programmed desorption of ammonia [45], or titration with bases [46]. Determination of acid sites in porous solid catalysts for the aqueous phase reactions using potentiometric titration method [47] showing several pK_a_ values. Presence of different sites and as a consequence utilization of non-Langmurian adsorption isotherms [47,48] while resulting in deviations of a classical Nernstian behavior, cannot, however, explain an increase of zeta potential or maxima with an increase of pH.

A more feasible explanation was advanced by Nosrati et al. [12], who noticed an increase of zeta potential with a pH increase from 3 to ca. 4.5 in the case of muscovite suspensions and a subsequent decrease above the latter value. Such behavior can be ascribed to leaching of multivalent metal ions (Al(III)), subsequent hydrolysis and specific adsorption and precipitation onto the solid surfaces. In addition to liberation of Al(III) and its subsequent contribution to the positive charge of the surface after readsorption, Al(III) at higher concentrations can also contribute to ionic strength. Qualitative description of the zeta potential behavior in the case of zeolites should thus take into account pH dependent speciation changes and leaching of alumina species. In fact, stability of zeolites at low pH has been elucidated in the literature. In particular, for acid leached beta zeolites it was shown in [49] that the ratio of Si/Al ratio increases with the severity of the treatment because both framework and extra-framework Al atoms are progressively leached from the zeolite.

Changes in the ionic strength of the solution during measurements can also happen because of ion exchange with alkali ions present even in the proton forms of commercial zeolites. In the current work, however, the ammonium forms were used for catalyst preparation, thus presence of alkali atoms did not influence the observed zeta potential behavior.

In general, experimental data presented above clearly demonstrate a very complex behavior of zeta potential for the studied materials. The current work was aimed at collection of reliable experimental data for zeolitic materials with different structure and acidity rather than providing a detailed theoretical explanation for the observed behavior. From the phenomenological observations, it became clear that in the case of zeolites the developed surface charge or zeta potential cannot be predicted as an additive value for silica and alumina zeta potentials corrected for the corresponding SiO_2_/Al_2_O_3_ ratio. This in practice implies that for preparation of supported catalysts by, for example, depositing a metal containing precursor by adsorption, a certain procedure operative for one type of zeolite with a particular acidity might not be valid for other materials. Metal deposition should be done in a way that the metal precursor will not be repelled from the surface. Zeta potential measurements performed in the current work clearly showed that the silica-to-alumina ratio, zeolite type, as well as temperature significantly influence the values of zeta potential, while concentration of solids in the slurry in the studied range of concentrations was less important.

Similar statements can be also made regarding catalyst shaping when the surface charge should be taken into account while developing experimental procedures for forming catalyst granules by e.g., extrusion. It can be at the same time argued how relevant are zeta potential measurements for catalyst shaping, in particular, extrusion occurring in high density suspensions. For example, the work of Kraushaar-Czarnetzki and co-workers, showing a possibility to control the aggregation and peptization of zeolites [6], was done in diluted suspensions. Repulsive interparticle forces induced from the electrostatic double layer can be accurately described by DLVO theory accounting for colloidal interactions between charged particles [50]. Zeta potential and the isoelectric point are thus relevant in describing the charge on the particle surface. In the case of suspensions with high concentration of solids, other types of adhesion forces especially in the presence of polymeric binders might be of more relevance.

## 4. Materials and Methods

### 4.1. Catalytic Materials

Several different commercial NH_4_-zeolites purchased from Zeolyst International (Conshohocken, Pensylvania, USA) with varying SiO_2_/Al_2_O_3_ ratio were used in this work, such as NH_4_-beta-25, NH_4_-beta-150 and NH_4_-beta-300. The last number in the zeolite labels denotes SiO_2_ to Al_2_O_3_ ratio. The NH_4_-zeolites were calcined using a stepwise temperature program: 25–250 °C (40 min)–400 °C (140 min) resulting in the proton form of zeolites. All materials were sieved below 32 μm fraction. Vista as well as silica gel Si-60 from Merck (Darmstadt. Germany) and fumed silica (Aldrich, Espoo, Finland) were applied. Al_2_O_3_ (Versal alumina GL25) was purchased from La Roche (Welwyn Garden City, UK). In addition, a material exhibiting an amorphous structure using a synthesis time of 24 h was prepared following the procedure described in [51] for comparison. It was denoted as Beta-22.

### 4.2. Characterization Methods

Prior to the zeta potential measurements, the supports: Al_2_O_3_, SiO_2_, and H-beta-25, H-beta-150, H-beta-300 zeolites were characterized using X-ray powder diffraction (XRD) for phase purity and structure, scanning electron microscopy (SEM) for morphology, nitrogen physisorption for specific surface area, particle morphology and periodicity. FTIR using pyridine as a probe molecule was performed to elucidate presence, strength and amount of Brønsted and Lewis acid sites. Elemental analysis was carried out using energy dispersive X-ray (EDAX) micro-analysis.

Pyridine (>99.5%) adsorption-desorption was studied using an ATI Mattson FTIR (LabX, Midland, ON, Canada). Self-supported wafers with the weight of 15–20 mg were prepared. Pyridine was adsorbed at 100 °C for 30 min. Thereafter a sample was evacuated at three different temperatures of 250 °C, 350 °C, and 450 °C. Spectra were recorded in vacuum with a spectral resolution of 2 cm^−1^. Spectral bands at 1540 cm^−1^ and 1450 cm^−1^ were used to identify Brønsted and Lewis acid sites. For the quantitative analysis, the extinction coefficients of Emeis [52] were applied.

Solid state ^27^Al-NMR spectra of H-beta-25, H-beta-150, H-beta-300 zeolite catalysts were used to analyse the location of aluminum species in the framework and extra framework positions.

Single pulse excitation spectra were recorded on an AVANCE III-800 spectrometer (Bruker, Germany) in 18.8 T magnetic field (^27^Al resonance frequency 208.4 MHz) using a Bruker MAS probe for 3.2 mm on zirconia rotors. The spinning speed of the samples was 22.0 kHz in all the experiments. To keep quantitative intensities 10° short excitation pulses were used at rf field strength 50 kHz and 0.1 s relaxation delay between the accumulations. Intensity has been normalized by dividing the absolute intensity by the mass of a sample and by a number of scans. The spectra were referenced to the resonance frequency of KAl(SiO_4_)_2_·12H_2_O.

### 4.3. Zeta Potential Measurements

The zeta potential measurements were performed with a Zetasizer Nano ZS (Malvern Instruments, Malvern, United Kingdom) using light scattering technique while for pH measurements the potentiometric method (MPT-2) was applied. The sample concentration was varied from 2 to 4 mg/mL suspended in de-ionized water. In the majority of measurements NaOH and HCl were used as titrants with no addition of indifferent electrolytes. In some measurements KCl was applied to change the ionic strength. Zeta potential was measured in the temperature range of 25–65 °C, and each measurement was repeated three times. The final zeta potential vs. pH curves were averaged from three measurements. Few tests were initially done using an equilibration procedure keeping the suspension for 24 h prior to measurements. Since no big differences were seen between this procedure and an accelerated one the majority of measurements were done with the latter approach. In essence, each time when a suspension was prepared the measurements started few minutes after that. The measurement time covering the whole pH range typically took one hour or slightly more depending on the sample. The Smoluchowsky theory was applied in determination of the zeta-potential.

## 5. Conclusions

A systematic investigation of zeta potential as a function of solution pH was made in the current work for H forms of beta zeolites exhibiting three different SiO_2_/Al_2_O_3_ ratios. The zeta potentials curves were measured in the 25–65 °C interval using silica and alumina as reference materials. To get statistically and physically reliable results, zeta potential was measured three times at every pH for the same sample altering also the concentration of solids in the slurry. Preliminary experiments showed a good agreement with the literature data published for alumina and silica.

The results for zeta potential measurements for H-beta zeolites showed that higher temperature of the solution decreased the zeta potential. Typically, there were maxima of the zeta potential values for highly acidic H-beta at a certain pH, not reported previously in the literature. For two zeolites, exhibiting the lowest Si/Al ratios, namely H-beta-25 and H-beta-150, the zeta potential curves were quite close to each other. These materials had also similar acidity. On the other hand, for low acidic H-beta-300 the zeta potential values were negative, while the shape of the zeta potential curve resembled that of silica and alumina.

Thus, there are several factors affecting the electrophoretic behavior of zeolites. An increase in alumina content leads to decreasing acidity (mobility) of bridging protons, but the number of acidic sites increases; therefore, zeta potential becomes a non-monotonic function of pH. Particle and pore sizes, S_BET_ value influence the amount of the bound water possessing lower activity as a solvent that can affect the mobility of protons and the structure of the electric double layer (EDL), i.e., zeta potential value. In other words, at the same structure of a surface in pores and at a surface of nano- and microparticles, the EDL structure should be different. External factors (T, content, pH) can differently affect the EDL structure in pores of different sizes or at surface of particles of different sizes.

## Figures and Tables

**Table 1:** Specific surface areas of catalysts studied for zeta potential measurements.

Catalysts	Specific Surface Area, (m^2^/g)	Ref.
H-Beta-25	807	[37]
H-Beta-150	664	[37]
H-Beta-300	805	[37]

**Table 2:** Determination of Brønsted and Lewis acid sites in H-beta zeolite catalysts.

Catalysts	Brønsted Acidity, (µmol/g)			Lewis Acidity, (µmol/g)			Ref.
	250 °C	350 °C	450 °C	250 °C	350 °C	450 °C	
H-beta-25	219	187	125	82	43	25	[39]
H-beta-150	176	161	72	43	23	10	[37]
H-beta-300	54	49	23	28	9	4	[39]

**Table 3:** Line intensities in ^27^Al-MAS NMR spectra from H-beta-25, H-beta-150, H-beta-300 zeolites.

Catalysts	Al(IV)a	Al(IV)b	Sum Al(IV)	Al(V)	Al(VI)a	Al(VI)b	Sum Al(VI)	Sum
H-beta-25	1.9	15.5	17.4	6.4	0.9	11.2	12.1	35.9
H-beta-150	3.8	15.1	18.9	6.8	0.5	11.9	12,4	38.2
H-beta-300	1.5	2.7	4.2	2.9	0.1	1.8	1.9	9.0

Al(IV)a and Al(IV)b denote aluminum in tetrahedral sites at 58 and 55 ppm, respectively.

**Table 4:** Zeta potential values for beta zeolites.

Material	Concentration (mg/mL)	T (°C)	Maximum Zeta Potential (mV) at pH as ( ) *	IEP 1 *	IEP2 *
H-beta-25	2–4	25	16 (4.5)	4.0	5.7
	2–4	50	0.7 (3.9)	3.7 **	4.0 **
	2–4	65	–3.7 (3.7)	no	
H-beta-150	2–4	25	14 (4.7)	4	5.7
	2–4	50	–3 (3.9)	no	
H-beta-150	2–4	65	–4.8 (3.6)	no	
H-beta-300	2–4	25	no	no	
	2–4	50	no	no	
	2–4	65	no	no	

* Average for concentrations 2–4 mg/mL; ** These values correspond to concentration of 2 mg/mL. No IEP were obtained for other concentrations.
